# Spontaneous Regression of Hepatocellular Carcinoma From Autoinfarction and Implications on Liver Transplantation

**DOI:** 10.14309/crj.0000000000000825

**Published:** 2022-07-12

**Authors:** Kevin Singh

**Affiliations:** 1 Department of Medicine, Northwell Health, Forest Hills, NY; 2 Department of Medicine, Erie County Medical Center, Buffalo, NY

## Abstract

Hepatocellular carcinoma (HCC) is the sixth most common cancer worldwide. Spontaneous regression of HCC due to autoinfarction is rare. This case series describes 2 cases of HCC autoinfarction that affected transplant candidacy: 1 patient previously ineligible because of tumor size and not meeting the Milan criteria became eligible after autoinfarction and tumor shrinkage while the second one was delisted in the view of improved symptoms of chronic liver disease and significant HCC regression. These cases provide an opportunity to review the pathogenesis of HCC autoinfarction and to remind practitioners of how this entity might alter decision-making around transplant eligibility.

## INTRODUCTION

Hepatocellular carcinoma (HCC), the sixth most common cancer worldwide, often requires liver transplantation as a definitive cure.^1^ The Milan criteria are most frequently used to determine liver transplantation eligibility in patients with HCC, with patients eligible if they have a solitary HCC of ≤5 cm or 2–3 HCC lesions no larger than 3 cm in size.^2^ Rarely, HCC can undergo autoinfarction or spontaneous regression and consequently change eligibility for liver transplantation. This case series describes 2 patients with HCC who underwent autoinfarction, which influenced their transplant eligibility. Describing these cases also provides an opportunity to review the pathogenesis and diagnosis of HCC autoinfarction and to remind practitioners of this entity and how it might alter consequent clinical decision-making regarding transplant eligibility.

## CASE REPORT

### Patient 1

**Figure 1.**
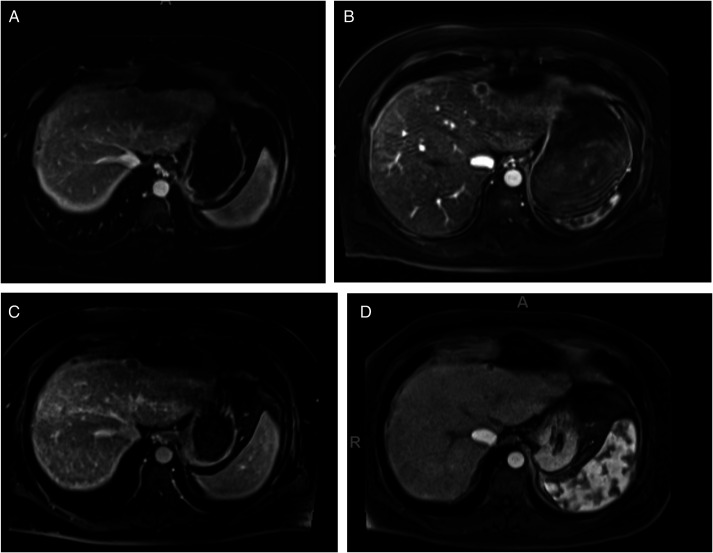
Imaging findings in patient 1: (A) gadolinium-enhanced magnetic resonance imaging (MRI) showing a new 1.5 × 1.3 cm LI-RADS 4 lesion in segment 2/4A. (B) Surveillance MRI 3 months later revealing an increase in size to 1.7 × 1.4 cm with arterial phase hyperenhancement and contrast washout on the venous phase (not shown) compatible with hepatocellular carcinoma (HCC) (LI-RADS 5). (C) A surveillance MRI performed 9 months after diagnosis of the liver lesion showed stable size and loss of arterial phase enhancement. (D) MRI 15 months after initial diagnosis of the liver lesion showing a reduction in size to 0.9 × 0.9 cm.

A 43-year-old man with alcohol-associated cirrhosis (initial model for end-stage liver disease [MELD] at listing was 16), type 2 diabetes, and hypertension underwent HCC screening with gadolinium-enhanced magnetic resonance imaging (MRI). Imaging revealed a new 1.5 × 1.3 cm lesion in segment 2/4A without arterial phase enhancement and venous phase contrast washout, which was classified as a LI-RADS 4 lesion (probably HCC; Figure 1). A surveillance MRI 3 months later revealed enlargement of the lesion to 1.7 × 1.4 cm with early arterial phase enhancement, compatible with HCC (LI-RADS 5; Figure 1). His serum alpha-fetoprotein (AFP) level was <2.00 (normal <8.78 ng/mL). Because the patient was already listed for liver transplantation and the HCC met the Milan criteria, further treatment was not pursued. Surveillance MRIs at 6-month intervals revealed loss of arterial phase enhancement and no change in lesion size, but then, further reduction to 0.9 × 0.9 cm was suggestive of HCC autoinfarction (Figure 1). The patient's clinical status improved after long-term alcohol abstinence (MELD score 8 without HCC exception points and resolution of ascites without the need for diuretics) and the HCC had regressed, so he was delisted. Six months and a year after delisting, the lesion decreased further in size to 0.7 and 0.7 cm and subsequently 0.6 × 0.6 cm.

### Patient 2

**Figure 2.**
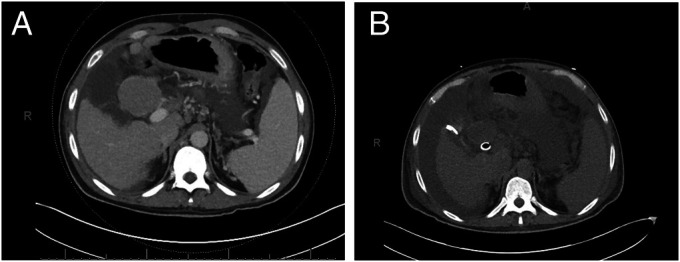
Imaging findings in patient 2: (A) gadolinium-enhanced magnetic resonance imaging showing a new 5.2 × 4.8 cm segment 4 lesion (LI-RADS 5). (B) A triple-phase computed tomography scan performed 1 month after initial diagnosis showed a reduction in size to 4.5 × 3.2 cm, suggestive of autoinfarction.

A 54-year-old man with decompensated cirrhosis due to hepatitis B with ascites, hepatic encephalopathy, esophageal varices, and HCC (found in segment 7 and treated successfully with transarterial chemoembolization 4 months earlier) underwent HCC surveillance with gadolinium-enhanced MRI that revealed a new, 5.2 × 4.8 cm segment 4 lesion (LI-RADS 5; Figure 2). The serum AFP was 4.3. One month later, a triple-phase computed tomography scan showed tumor shrinkage to 4.5 × 3.2 cm, suggestive of autoinfarction (Figure 2). The patient's MELD score was 37, and after tumor regression, the remnant HCC lesion met the Milan criteria, allowing listing for liver transplantation. Four weeks after listing, the patient underwent transplantation and examination of the explanted liver revealed no lesions such as viable HCC. Five years after transplantation, no HCC recurrences had been detected on screening.

## DISCUSSION

HCC autoinfarction is rare, with an estimated incidence of 1 case in 60,000–100,000 patients with HCC worldwide.^3,4^ HCC autoinfarction has been reported in both pretransplant and posttransplant populations but most frequently in male patients, Southeast Asians, and patients with chronic liver disease associated with alcohol abuse and hepatitis B or C infection.^5^ Radiographic or histopathologic evidence of tumor involution, such as reduction in size or complete tumor regression in patients who have not had recent therapeutic interventions, suggests a diagnosis of HCC autoinfarction.^6^ The exact cause of autoinfarction remains unknown, but the main proposed mechanisms of autoinfarction include tumor hypoxia or immunologic factors (Table 1).^5^

**Table 1.** Proposed mechanisms for autoinfarction of hepatocellular carcinoma

Tumor hypoxia	Immunologic factors
Massive gastrointestinal hemorrhage	Smoking abstinence
Surgery	Alcohol abstinence
Rapidly growing tumors	Prolonged fever
Hepatic angiography	Vitamin K
Hemodialysis	Herbal medications
Portal vein thrombosis	Antidiabetic medications
Hepatic artery thrombosis	
Hepatic arterioportal shunt	

Tumor regression can influence the clinical management of patients with HCC, as seen here. The first patient had underlying alcohol-associated cirrhosis and was listed for transplantation before autoinfarction. Long-term alcohol abstinence likely led to improvements in background cirrhosis, HCC regression, and consequent delisting from the transplantation list because of the clinical improvement.^5,7–10^ The patient did not undergo biopsy, but although hepatic adenomas and focal nodular hyperplasia may have similar presentations to HCC and the radiographic features of HCC and regenerative or dysplastic nodules are similar, the presence of a pseudocapsule and contrast washout during the portal venous phase ruled out these lesions in the first patient.^11^ By contrast, the second patient was initially diagnosed with an HCC that did not meet the Milan criteria for transplantation because of its size, and it would have required locoregional therapies for downstaging. However, shortly after the HCC diagnosis, the tumor shrunk and then met the Milan criteria, allowing for transplantation without the need for locoregional therapies. Other criteria for larger lesions have been proposed including the University of California, San Francisco criteria for a single lesion not exceeding 6.5 cm or 2–3 lesions less than 4.5 cm with total tumor diameter not exceeding 8 cm, but their use remains experimental.^12,13^

The underlying reason for autoinfarction was unclear in the second patient, although it is possible that the tumor regressed because of it outgrowing its blood supply. In 30% of HCC cases, AFP levels are normal—as seen here in both patients—and may remain low even in advanced HCC.^14^

Ultrasound is the recommended screening modality for HCC, mainly because of its low cost, although the effectiveness of ultrasound is limited by operator skill; indeed, in 20% of cases, early HCC lesions may not be detected, especially in patients with obesity and in livers with coarse echotexture.^15,16^ In this study, MRI was used to complement ultrasound because of its higher sensitivity for detecting HCC, especially for lesions <2 cm. In a recent study from a tertiary transplant center, MRI was used to screen for HCC in over half of the patients, so its use is starting to become standard.^17^ Nevertheless, lesions may still be misclassified on screening MRI, so caution should be exercised in this context.^18^

Because autoinfarction is rare, when detected, clinicians should continue to monitor tumor changes with serial imaging because tumor shrinkage could influence management, especially in patients previously listed for transplantation who may no longer require it or patients who had been disqualified for transplantation based on eligibility criteria.

## DISCLOSURES

Financial disclosure: None to report.

Informed consent was obtained for this case report.
